# Beyond Poiseuille: Preservation Fluid Flow in an Experimental Model

**DOI:** 10.1155/2013/605326

**Published:** 2013-08-26

**Authors:** Saurabh Singh, Lucy V. Randle, Paul T. Callaghan, Christopher J. E. Watson, Chris J. Callaghan

**Affiliations:** ^1^University Department of Surgery, Addenbrooke's Hospital, NIHR Comprehensive Biomedical Research Centre, Cambridge CB2 0QQ, UK; ^2^School of Chemical and Physical Sciences, Victoria University of Wellington, Wellington, New Zealand; ^3^Department of Renal Surgery, Guy's Hospital, London SE1 9RT, UK

## Abstract

Poiseuille's equation describes the relationship between fluid viscosity, pressure, tubing diameter, and flow, yet it is not known if cold organ perfusion systems follow this equation. We investigated these relationships in an *ex vivo* model and aimed to offer some rationale for equipment selection. Increasing the cannula size from 14 to 20 Fr increased flow rate by a mean (SD) of 13 (12)%. Marshall's hyperosmolar citrate was three times less viscous than UW solution, but flows were only 45% faster. Doubling the bag pressure led to a mean (SD) flow rate increase of only 19 (13)%, not twice the rate. When external pressure devices were used, 100 mmHg of continuous pressure increased flow by a mean (SD) of 43 (17)% when compared to the same pressure applied initially only. Poiseuille's equation was not followed; this is most likely due to “slipping” of preservation fluid within the plastic tubing. Cannula size made little difference over the ranges examined; flows are primarily determined by bag pressure and fluid viscosity. External infusor devices require continuous pressurisation to deliver high flow. Future studies examining the impact of perfusion variables on graft outcomes should include detailed equipment descriptions.

## 1. Introduction

Adequate organ preservation is essential to subsequent graft function and is therefore fundamental to successful organ transplantation. Despite the introduction of machine perfusion [1–6], the overwhelming majority of organ procurements still take place with standard techniques using intravascular instillation of cooled preservation fluids and cold storage. However, although preservation fluids have been investigated extensively [7–10], comparatively little research has been carried out on the optimal method of delivering them [11].

Preservation fluid pressure appears to influence graft function [12–17], but it is also likely that fluid flow rate is clinically important, as heat transfer, and therefore organ cooling, is flow-dependent [18], and rapid organ cooling has been shown to improve cellular and organ viability [19, 20]. These two variables are interrelated, and many clinicians would assume that Poiseuille's equation adequately describes this relationship. Poiseuille's equation states that fluid flow rate (*Q*) through a tube is inversely proportional to tube length (*L*) and fluid viscosity (*μ*) and is proportional to the pressure drop across the tube (Δ*P*) and the tube radius (*r*) to the fourth power [21]:

(1) $$\begin{array}{c} Q=\frac{\Delta P\pi r^{4}}{8\mu L}. \end{array}$$

However, Poiseuille's equation only applies to fluids with a constant viscosity regardless of the fluid velocity (known as Newtonian fluids), with nonturbulent flow and a no-slip boundary condition (i.e., the fluid immediately adjacent to the tubing wall is stationary). As University of Wisconsin (UW) solution is thought to be a non-Newtonian fluid [22] and plastic tubing may lead to fluid “slipping” (i.e., the fluid adjacent to the tubing wall is mobile), the equation may not be able to accurately predict how alterations to tubing length, fluid viscosity, cannula size, and bag pressure affect fluid flow. 

We therefore used a perfusion model to determine whether Poiseuille's equation can adequately describe this system and if clinically relevant alterations have a significant impact on perfusate flow. We aimed to provide a sound basis for selecting perfusion equipment and to establish a rationale for *in vivo* studies to address these issues further.

## 2. Materials and Methods

### 2.1. Viscosity Measurement

Fluid viscosity was measured under non-slip conditions at varying strain rates using an ARES rheometer (TA Instruments New Castle, DE, USA). ThickenUp Clear (Nestlé, Croydon, UK) was used at a concentration of 0.2 g per 100 mL water. Tests were carried out using Couette (cup and bob) geometry at a temperature of 7°C. The range of strain rates reflected those present in the perfusion model.

### 2.2. Perfusion Model

Standard equipment and fluids currently used for organ procurement by the Cambridge Transplant Unit were utilised. One-litre bags of UW solution (ViaSpan, Bristol-Myers Squibb Pharma, Garden City, NY, USA) or Marshall's hyperosmolar citrate (HOC; Soltran, Baxter Healthcare, Thetford, UK) were attached to a Y-connector irrigation set (Fast-Flow, Baxter S.A., Lessines, Belgium) of length 220 cm with internal diameter 6 mm. This was connected to a 300 cm length of Flexi-Rib tubing (Pennine Healthcare, Derby, UK) of internal diameter 7 mm; in some experiments a 150 cm length was used. Finally, this was attached to one of four cannulas (14, 16, 18, or 20 Fr, Terumo, Leuven, Belgium). The cannula was held horizontally and fluid was collected in a measuring cylinder. 

Pressure on the preservation fluid was gravity alone (0.4 m or 0.8 m) or gravity plus additional external pressure (100 mmHg applied continuously while the bag emptied or 100 mmHg initially only). Initial external pressure was provided by a 1000 mL pressure infusion sleeve (XL Group, Dallas, TX, USA) inflated to 100 mmHg. Continuous external pressure was applied by a C-Fusor 1000 mL pressure infusor (Smiths Medical, Dublin, OH, USA) maintained at 100 mmHg by a pressure PAC compressor (Smiths Medical, Dublin, OH, USA). Both systems were calibrated via pressure monitoring tubing and a transducer (Edwards Lifesciences, Irvine, CA, USA) attached to a Datex-Ohmeda S/5 critical care monitor (GE Healthcare, Chalfont St. Giles, UK).

After priming the system with preservation fluid, a tubing clamp was placed on the Flexi-Rib tubing. After releasing the clamp, fluid was collected in a measuring cylinder. The time for the first 500 mL of perfusate to flow was measured using a stopwatch. Three measurements were taken, and mean and SD flow rates were calculated. 

As preservation fluid viscosity varies with temperature [23], this was kept constant by storing fluid bags in an ice box before use and placing the measuring cylinder in an ice bucket. The temperature of perfusate in the measuring cylinder was recorded using an electronic thermometer (Hanna Instruments, Woonsocket, RI, USA). 

### 2.3. Data Analysis and Statistics

In order to find the average effect of changing a single variable across all combinations, percentage change in flow rate was calculated for each pair of experiments (i.e., before and after the change of variable), and the mean (SD) percentage changes were determined. Statistical significance was determined using a two-tailed paired Student's *t*-test (GraphPad Prism 5.02 for Windows, GraphPad Software, San Diego, CA, USA); *P* < 0.05 was considered significant.

## 3. Results

### 3.1. Perfusate Viscosity

In order to determine if Marshall's HOC and UW are Newtonian fluids, viscosity was measured at varying velocities (expressed as strain rates). Water and ThickenUp Clear were controls; water is a Newtonian fluid, and ThickenUp Clear is a xanthan gum-based thickening agent known to be non-Newtonian. 

**Figure 1:**
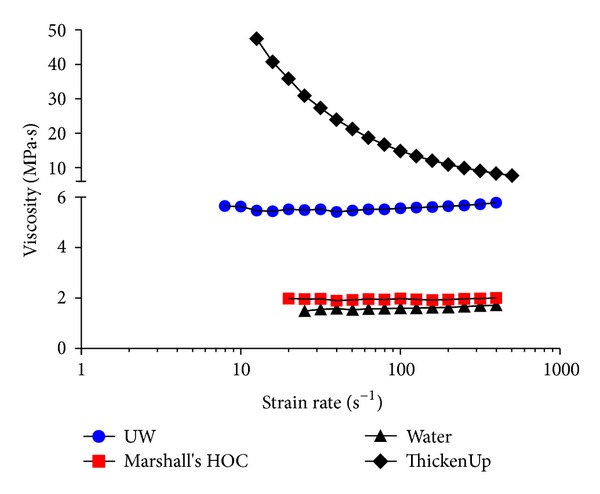
Graph showing the viscosities of UW, Marshall's HOC solution, water, and ThickenUp Clear over a range of strain rates. Strain rates are plotted on a log scale, and viscosity is plotted on an interrupted axis to enable all values to be shown. Temperature was kept constant at 7°C.

Figure 1 shows that Marshall's HOC has a viscosity similar to that of water, but UW has almost three times the viscosity of both. The viscosities for both Marshall's HOC and UW fluids were constant over the strain rates examined and therefore appear to be Newtonian fluids, contrary to previous reports [22]. 

### 3.2. Perfusion Model Measurements

**Figure 2:**
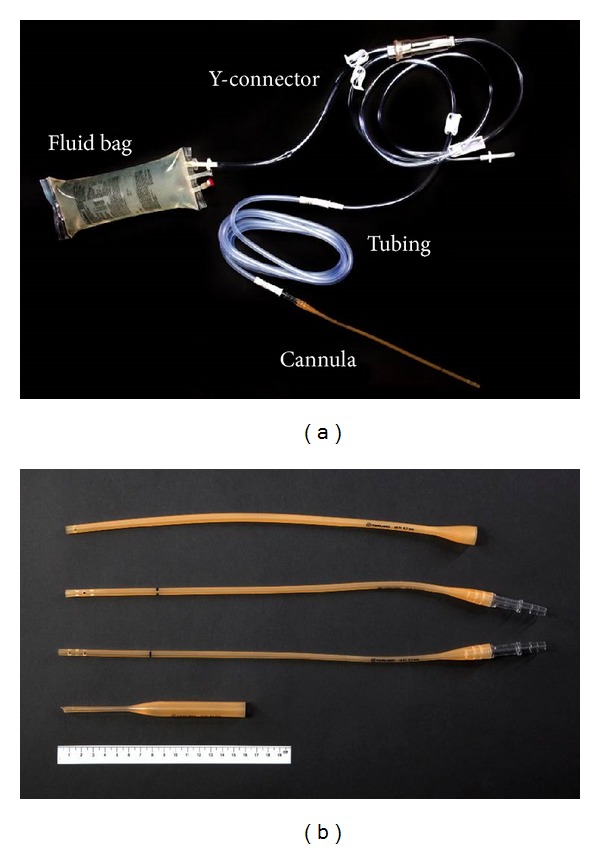
(a) Photograph showing a UW bag with Y-connector, Flexi-Rib tubing, and an 18 Fr cannula. (b) Cannulas used in the study (from top to bottom, 20, 18, 16, and 14 Fr, resp.). A 20 cm ruler is present for scale.

Flow using 96 different combinations of cannula size, tubing length, fluid type, and bag infusion pressure was measured (Figures 2(a) and 2(b)). Measurements were repeated three times for each combination. Perfusate temperature was kept constant, with a mean (SD) temperature of 7 (1)°C.

**Figure 3:**
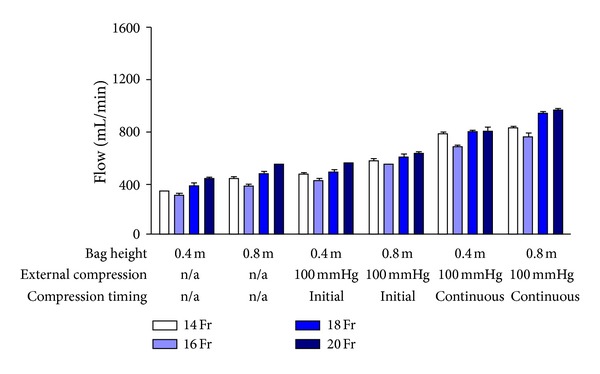
Graph showing the relationship between cannula size and bag pressure on mean (SD) UW flow. Connecting tubing length was kept constant at 3 m.

**Figure 4:**
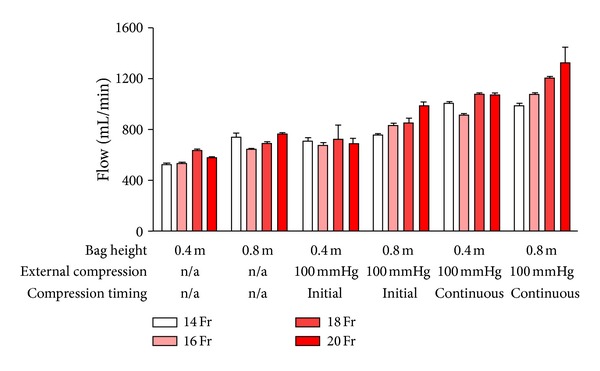
The impact of cannula size and bag pressure on mean (SD) Marshall's HOC flow. Connecting tubing length was kept constant at 3 m.

Increasing the cannula size generally increased flow, regardless of the effect of other variables. Figures 3 and 4 show flow rates for UW and Marshall's HOC through cannulas of different sizes and at different pressures when tubing length was 3 m. These findings were similar to those when tubing length was halved (data not shown). When all other variables were kept constant, changing from a 14 Fr to a 20 Fr cannula increased flow by a mean (SD) of 13 (12)% (*P* < 0.001). Unexpectedly, flow through a 16 Fr cannula was lower than that through a 14 Fr cannula with a mean (SD) drop of 9 (10)% (*P* < 0.001). This effect seemed to be more marked for UW than Marshall's HOC (mean (SD) drop of 15 (9)% versus 4 (8)%; *P* = 0.002).

We hypothesised that the increased flow through a 14 Fr cannula was either due to its shorter length compared to that of a 16 Fr cannula, or related to turbulence caused by the three pairs of side holes at the 16 Fr cannula tip (Figure 2(b)). To test these hypotheses, the flow of UW at 0.8 m height was measured through full length tubing and a 16 Fr cannula; mean flow was 376 mL/min. The side holes were then removed by cutting off the distal 2.5 cm of the cannula, leading to a mean flow of 436 mL/min. After shortening the 16 Fr cannula by a further 20 cm (i.e., to the same length as a 14 Fr cannula) mean flow was 489 mL/min. Therefore, both the length and the side holes of the 16 Fr cannula contribute to reduced flows. 

**Figure 5:**
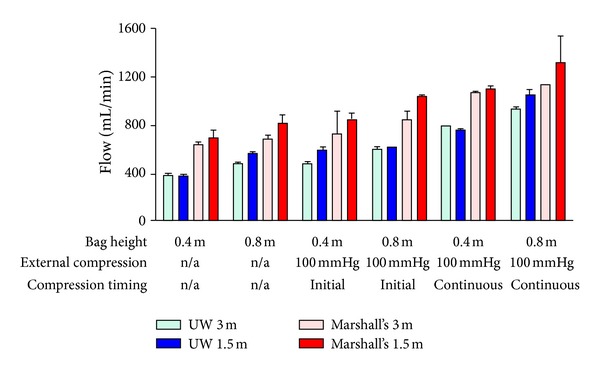
The impact of connecting tubing length on UW and Marshall's HOC flow (mean and SD) over a range of bag pressures. Data shown is for a system with a cannula size of 18 Fr.

When comparing all combinations of fluid, cannulas, and bag pressures, reducing the tubing length from 3 to 1.5 m increased fluid flow by a mean (SD) of 11 (10)% (*P* < 0.001).  Figure 5 shows data from an experiment using an 18 Fr cannula, but the pattern of flows with other cannula sizes was similar (data not shown). 

Flows were significantly lower with UW than with Marshall's HOC (Figure 5). Replacing UW with Marshall's HOC increased fluid flows by a mean (SD) of 45 (17)% (*P* < 0.001), even though the viscosity of Marshall's solution is three times lower than UW.

Finally, the effect of increasing bag pressure was determined. As expected, increased bag height augmented fluid flow, and continuous external pressure improved fluid flows more than initial external pressure alone (Figures 3–5). Doubling the hydrostatic pressure by elevating the bag from 0.4 to 0.8 m led to a mean (SD) increase of 19 (13)% (*n* = 48 pairs; *P* < 0.001), rather than a doubling in flow as expected from Poiseuille's equation. When external pressure bags were used, continuous pressure at 100 mmHg increased flow by a mean (SD) of 43 (17)% when compared to 100 mmHg pressure applied initially only (*n* = 32 pairs; *P* < 0.001). 

## 4. Discussion

This study has demonstrated that Poiseuille's equation does not adequately describe the relationships between system variables in a perfusion model, and that subtle changes to cannula length or design may have unexpectedly significant impacts on flow. Previous studies have investigated flow properties of ureteric stents [24] and intravenous cannulae [25], but the relative impact of fluid type, tubing length, and duration of external pressurisation was not examined. To our knowledge, this is the first description of flow characteristics in a large-diameter preservation fluid system. 

Poiseuille's equation predicts that doubling pressure, or halving fluid viscosity, would double perfusate flow. However, these relationships only hold true for nonturbulent Newtonian fluid flow where no-slip boundary conditions exist (i.e., the fluid immediately adjacent to the tubing wall is stationary). We have clearly demonstrated that doubling the pressure results in only a modest increase in flow, and that, although both Marshall's HOC and UW solutions are Newtonian fluids, their relative viscosity differences are not reflected in their respective flow rates. 

The presence of turbulence can be predicted by calculating the Reynolds number (i.e., (fluid density × fluid velocity × tube diameter)/fluid viscosity); turbulence is certain with a Reynold's number above 4000. Calculations using data from all experimental combinations resulted in Reynold's numbers <2500, and therefore turbulence can reasonably be excluded as a reason for why the model does not obey Poiseuille's equation. Slipping of preservation fluid adjacent to the plastic tubing wall is therefore the likely explanation for our findings. The slip phenomenon is difficult to predict and is dependent on both the flow rate and the physicochemical properties of the tubing wall and fluid. The unpredictable nature of fluid flow is exemplified by the adverse influence of side holes on fluid flow through the 16 Fr cannula. Both clinicians and cannula manufacturers should be aware that subtle changes in cannula design may have unexpected impacts on function. 

Of the variables examined, perfusate pressure and fluid type had most influence on flow. Low viscosity Marshall's increased flow by an average of 45% when compared to colloid-rich, high-viscosity UW. This is similar to the findings of Kay et al. who described UW flow rates half that of Marshall's, though they used a small-diameter blood-giving set rather than a larger procurement perfusion set [18]. Perfusate pressure made the largest contribution to flow; doubling the hydrostatic pressure increased flows by an average of 19%, and adding continuous external pressure at 100 mmHg at the greater height more than doubled flow compared to 0.4 m height alone. The use of continuous rather than initial external bag pressurisation resulted in flows increasing by almost 50%. This finding is of particular interest given that previous papers investigating the clinical effect of high pressure perfusion have not described how the pressurisation was carried out [14–17]. 

Our model has a number of limitations. Firstly, we are unsure as to why our measurements of UW solution viscosity differ from those of van der Plaats et al. [22]. Regardless of this difference, van der Plaats' finding would similarly result in failure of the Poiseuille relationship. Secondly, pressure and flow are determined by resistance (Ohm's law), which is fixed in the nondistensible model used here but is likely to be more complex *in vivo*, as total resistance consists of the resistance both from the perfusion system and the donor vasculature. Donor resistance will vary with flow/pressure due to blood vessel distension and will be reduced when arterial branches are divided. Other variables expected to influence pressure/flow *in vivo* include organ size, presence of parenchymal and vascular disease, vessel diameter, dynamic constriction of arterioles in response to cold, venting technique, and the length of the clamped aortic segment. Given these complexities, it would be difficult and time consuming to perform accurate *in vivo* experiments. 

## 5. Conclusions

Our study provides the only available rationale for selecting perfusion equipment. Cannula size can largely be chosen on the basis of ease of cannulation rather than on perceived impact on flow. Likewise, tubing length should be determined by clinical considerations. If high flow is required, this is best achieved by using continuous bag pressurisation with a low viscosity perfusion fluid. In addition, this study highlights the inadequacy of our understanding of the optimal means of delivering preservation fluid. This may be critical in improving usage of marginal organs and preventing lesions associated with poor preservation, for example, ischaemic-type biliary strictures [16, 26]. Adequately powered clinical studies examining the impact of varying preservation pressure and flow should be conducted. Previous papers investigating the effect of preservation fluid pressure have not described the tubing lengths or cannula sizes used [14, 15, 17], and therefore at least some of the effects that they ascribe to pressure may be due to variations in the flow characteristics of the system used.

## Abbreviations

HOC: Hyperosmolar citrate
SD: Standard deviation
UW: University of Wisconsin.
